# An integrated telemedicine platform for the assessment of affective physiological states

**DOI:** 10.1186/1746-1596-1-16

**Published:** 2006-08-01

**Authors:** Christos D Katsis, George Ganiatsas, Dimitrios I Fotiadis

**Affiliations:** 1 Dept. of Medical Physics, Medical School, University of Ioannina, GR 45 110 Ioannina, Greece; 2 Unit of Medical Technology and Intelligent Information Systems, Dept. of Computer Science, University of Ioannina, GR 45 110 Ioannina, Greece

## Abstract

AUBADE is an integrated platform built for the affective assessment of individuals. The system performs evaluation of the emotional state by classifying vectors of features extracted from: facial Electromyogram, Respiration, Electrodermal Activity and Electrocardiogram. The AUBADE system consists of: (a) a multisensorial wearable, (b) a data acquisition and wireless communication module, (c) a feature extraction module, (d) a 3D facial animation module which is used for the projection of the obtained data through a generic 3D face model; whereas the end-user will be able to view the facial expression of the subject in real time, (e) an intelligent emotion recognition module, and (f) the AUBADE databases where the acquired signals along with the subject's animation videos are saved. The system is designed to be applied to human subjects operating under extreme stress conditions, in particular car racing drivers, and also to patients suffering from neurological and psychological disorders. AUBADE's classification accuracy into five predefined emotional classes (high stress, low stress, disappointment, euphoria and neutral face) is 86.0%. The pilot system applications and components are being tested and evaluated on Maserati's car. racing drivers.

## Background

The use of emotional understanding using computers is a field of increasing importance. In many ways emotions are one of the last and least explored frontiers of intuitive human computer interaction. This may be explained by the fact that computers are traditionally viewed as logical and rational tools, something that is incompatible with the often irrational and seeming illogical nature of emotions [1]. It is also apparent that we as humans, while extremely good at feeling and expressing emotions, still cannot agree on how they should best be defined [2].

After a century of research, there is little agreement about a definition of emotions and many theories have been proposed. A number of these could not be verified until recently when improved measurement of specific physiological signals became available. In general emotions are short-term, whereas moods are long-term, and temperaments or personalities are very long-term [3]. Furthermore, the physiological muscle movements, comprising what looks to an outsider to be a facial expression, may not always correspond to a real underlying emotional state.

Emotion consists of more than its outward physical expression; it also consists of internal feelings and thoughts, as well as other internal processes of which the person experiencing the emotion may not be aware. As machines and people begin to co-exist and cooperatively share a variety of tasks, the need for machines to constantly evaluate the affective condition of humans becomes more than apparent [4,5]. This has prompted researchers in the engineering and computer science communities to develop automatic ways for computers to recognise emotions. The labelling of emotions into different states led most researchers to use pattern recognition approaches for recognising emotions, using different modalities as inputs to the emotion recognition models. The work in automatic understanding of affective condition has focused on classification of the universal expressions (FACS) defined by Ekman [6]. These expressions are sadness, anger, fear, disgust, surprise, happiness, neutral and contempt. Thus, the implemented algorithms were tailored towards developing models to recognise the universal expressions from static images or video sequences [7-11]. These facial actions are essentially facial phonemes, which can be assembled to form facial expressions. There are also recent methods that employ a combination of audio and video signals for emotion recognition [12-18].

One of the hallmarks in emotion theory is whether distinct physiological patterns accompany each emotion [19]. Ekman et al. [20] and Winton et al. [21] provided some of the first findings showing significant differences in autonomic nervous system signals according to a small number of emotional categories or dimensions, but there was no exploration of automated classification. Flidlund and Izard [22] appear to be the first who applied pattern recognition (linear discriminants) on the classification of four different emotions (happiness, sadness, anger, fear) from physiological signals, attaining rates of 38–51 % accuracy. Similar efforts aimed at finding physiological correlates, focusing on t-tests or analysis of variance comparisons and combining data over many subjects, where each was measured for a relatively small amount of time [23,24]. Finally Picard et al. [4] classified physiological patterns for a set of eight emotions (including neutral) by applying pattern recognition techniques and focusing on felt emotions of a single subject over sessions spanning many weeks.

Although dealing with emotion recognition, the aforementioned techniques present the following limitations: (i) they are all materialized in laboratory environments therefore their effectiveness in real conditions is unknown, (ii) they are not real time and (iii) the data acquisition systems used for them are not wearable. The work in this paper is novel, since it presents a system that automatically monitors and classifies the psychological condition of human subjects from a set of emotions. The system is designed to be applicable to persons operating under extreme stress conditions, such as car-racing drivers. Medical applications are mainly based on the ability of supporting clinical diagnosis related to all the pathologies according to which the patient's capability to feel and express emotions is limited or totally absent.

## Materials and methods

When we are frightened, our heart races; our breathing becomes rapid; our mouth becomes dry; our muscles tense; and our palms may become sweaty. These bodily changes are mediated by the autonomic nervous system, which controls heart muscle, smooth muscle, and exocrine glands [25]. The autonomic nervous system itself can be divided into sympathetic and parasympathetic branches. Both operate in conjunction with each other and with the somatic motor system to regulate most types of behavior, whether in normal or emergency situations. Certain emotions may result in a wide variety of bodily reactions comparable to the ones described above. These bodily reactions can be monitored and measured. Our goal is to use these reactions and by means of special bio-sensors, to deduce the emotional state of the user.

AUBADE estimates the emotional state of human subjects by classifying vectors of features extracted from: Facial Electromyogram (EMG), Respiration, Electrodermal Activity (EDA) and Electrocardiogram (ECG).

### Electrodermal Activity (EDA)

It is also referred as skin conductance activity because of the underlying principle of measurement. EDA describes alterations – in skin's ability to conduct electricity – that occur due to interactions between environmental events and an individual's psycho-physiological state. More Specifically, it is related to sympathetic nervous system activity, which innervates the eccrine sweat glands; and has been associated with measures of emotion, arousal, and attention [26]. The EDA reading is typically characterized by two components: a tonic baseline level and short term phasic responses superimposed on the tonic baseline level. Phasic responses (momentary increases in skin conductance) determine the event-related responses that occur in an individual, due to environmental stimuli. A stimulus may be anything from a thought burst to a deep sigh. EDA is one of the fastest, most robust and well-studied physiological measures. It has been previously employed in assessing the difficulty of driving tasks [27]; in determining stress in anticipatory anxiety studies [28] and as part of lie detectors [29].

### Facial Electromyogram (EMG)

It refers to the muscle activity or frequency of muscle tension of a certain muscle. Muscle activity has been shown to increase during stress. People may unconsciously clench their muscles in a state of mental stress or fatigue even when no physical activity is required [30]. Firing from this muscle could indicate either unconscious clenching due to stress or firing due to motion.

### Electrocardiogram (ECG)

The ECG signal is the manifestation of contractile activity of the heart. Heart activity is a valuable indicator of the individual's overall activity level. For example heart rate accelerations occur in response to exercise, emotional states, loud noises, sexual arousal and mental effort [31]. Lower heart rate is generally associated with a relaxed state or a state of experiencing pleasant stimuli.

### Respiration

Respiration is an indicator of how deep and fast a person is breathing. Emotional excitement and physical activity are reported to lead to faster and deeper respiration [32]. Peaceful rest and relaxation lead to slower and shallower respiration. A state of stress would therefore be indicated by frequent respiration; however, sudden stressors such as startle tend to cause momentary cession of respiration.

**Figure 1:**
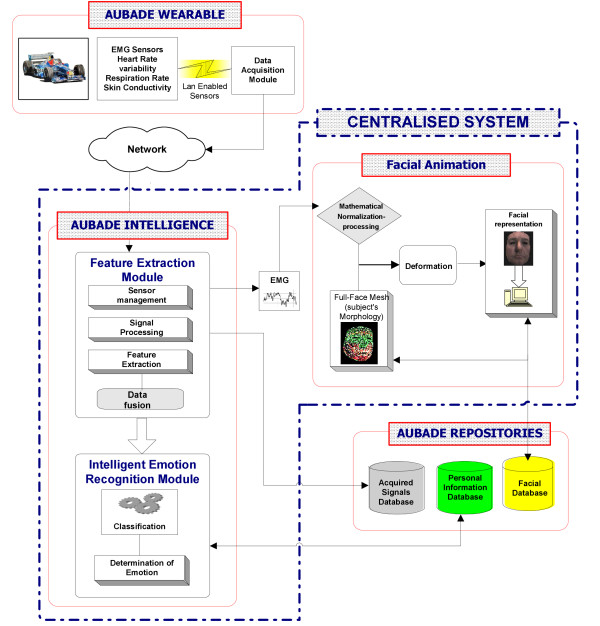
The AUBADE System Architecture.

AUBADE's development is based on the utilization of the latest technology advances in biosensors, medical wearable devices and systems, signal processing and decision support techniques, communication standards, security mechanisms, and facial muscle activity representation. The systems architecture is presented in Fig. 1. A detailed description of the AUBADE system's functionalities and modules follows:

#### a) The wearable module

It is a non-invasive, ergonomic, comfortable and easy to use wearable that includes a number of sophisticated bio-sensors gathering raw physiological data (facial EMG, Resp, EDA and ECG). The wearable is composed of three pieces: i) the mask containing sixteen EMG textile fireproof sensors, ii) the three-lead ECG and Respiration sensors on the thorax of the driver and iii) the EDA textile and fireproof sensor placed inside the drivers glove.

#### b) The data acquisition and wireless communication module

The signal acquisition unit consists of both hardware (data acquisition card) and software components. It appropriately collects, filters, pre-processes, formats and stores all biosignals obtained from the sensors of the wearable. The pre-processing procedure (sampling rate and filters used) is presented in Table 1. The resolution used during signal digitization is 12 bit. The data acquisition module is also responsible for controlling sensor behaviour and output and information feedback is provided for sensor operations.

**Table 1:** Biosignals Preprocessing

**Signal**	**Sampling Rate**	**Filters used**
Facial EMG (16 channels)	1000 Hz	Low pass (500 Hz)
ECG (3 channels)	500 Hz	Low pass (100 Hz)
EDA	50 Hz	Moving average
respiration	50 Hz	Moving average

AUBADE's wireless communication module is activated by the system end-user and is responsible for the secure transfer of the vital signs collected and processed by the Data Acquisition Unit. The user measurements are transferred through either the existing or a wireless LAN (Bluetooth or IEEE 802.11B) to the Centralised System for further analysis. Bluetooth is superior for medical applications based on the following properties:

*(i) range*: The range can vary from 1 m (Class 3) to 100 m (Class 1). No direct optical connection is necessary.

*(ii) bandwidth*: The bandwidth is up to 721 kbit/s in one direction. These values are theoretically sufficient for about 100 ECG channels and can be robustly attained even in a "noisy" environment by means of frequency hopping [33].

#### (c) The feature extraction module

The pre-processed biosignals are converted into vectors of extracted features that can be used by the Intelligent Emotion Recognition module in order to determine subject's basic emotions. The selected features provide a combination of simple statistics and complicated characteristics which are related to the nature of the physiological signals and the underlying classification problem. Furthermore, in this module sensor behaviour is also controlled; unseemly signals are not taken into account for processing and no features are extracted.

**Figure 2:**
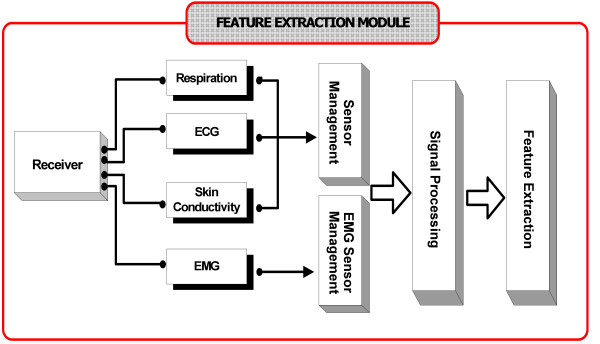
The Feature Extraction Module.

Fig. 2 presents a schematic representation of the module. Indicatively, some of the features are analyzed below:

### Mean and median frequency

They compute vectors of mean and median frequencies over time for a specific input signal.

**Means of the absolute values of the first and second differences **(mean_abs_fd and mean_abs_sd): For an acquired biosignal *X*_*N *_= (*x*_1_, *x*_2_,...,*x*_*N*_) the mean_abs_fd and mean_abs_sd are defined as:

mean_abs_fd=1N−1∑n=1N−1|xn+1−xn|,     (1)
 MathType@MTEF@5@5@+=feaafiart1ev1aaatCvAUfKttLearuWrP9MDH5MBPbIqV92AaeXatLxBI9gBaebbnrfifHhDYfgasaacH8akY=wiFfYdH8Gipec8Eeeu0xXdbba9frFj0=OqFfea0dXdd9vqai=hGuQ8kuc9pgc9s8qqaq=dirpe0xb9q8qiLsFr0=vr0=vr0dc8meaabaqaciaacaGaaeqabaqabeGadaaakeaacqWGTbqBcqWGLbqzcqWGHbqycqWGUbGBcqGGFbWxcqWGHbqycqWGIbGycqWGZbWCcqGGFbWxcqWGMbGzcqWGKbazcqGH9aqpdaWcaaqaaiabigdaXaqaaiabd6eaojabgkHiTiabigdaXaaadaaeWbqaamaaemGabaGaemiEaG3aaSbaaSqaaiabd6gaUjabgUcaRiabigdaXaqabaGccqGHsislcqWG4baEdaWgaaWcbaGaemOBa4gabeaaaOGaay5bSlaawIa7aiabcYcaSiaaxMaacaWLjaGaeiikaGIaeGymaeJaeiykaKcaleaacqWGUbGBcqGH9aqpcqaIXaqmaeaacqWGobGtcqGHsislcqaIXaqma0GaeyyeIuoaaaa@59C3@

mean_abs_sd=1N−2∑n=1N−2|xn+2−xn|,     (2)
 MathType@MTEF@5@5@+=feaafiart1ev1aaatCvAUfKttLearuWrP9MDH5MBPbIqV92AaeXatLxBI9gBaebbnrfifHhDYfgasaacH8akY=wiFfYdH8Gipec8Eeeu0xXdbba9frFj0=OqFfea0dXdd9vqai=hGuQ8kuc9pgc9s8qqaq=dirpe0xb9q8qiLsFr0=vr0=vr0dc8meaabaqaciaacaGaaeqabaqabeGadaaakeaacqWGTbqBcqWGLbqzcqWGHbqycqWGUbGBcqGGFbWxcqWGHbqycqWGIbGycqWGZbWCcqGGFbWxcqWGZbWCcqWGKbazcqGH9aqpdaWcaaqaaiabigdaXaqaaiabd6eaojabgkHiTiabikdaYaaadaaeWbqaamaaemGabaGaemiEaG3aaSbaaSqaaiabd6gaUjabgUcaRiabikdaYaqabaGccqGHsislcqWG4baEdaWgaaWcbaGaemOBa4gabeaaaOGaay5bSlaawIa7aiabcYcaSiaaxMaacaWLjaGaeiikaGIaeGOmaiJaeiykaKcaleaacqWGUbGBcqGH9aqpcqaIXaqmaeaacqWGobGtcqGHsislcqaIYaGma0GaeyyeIuoaaaa@59E5@

where *x*_*i *_denotes a signal sample and *N *is the number of samples. These features are approximations of the first and second derivate respectively and therefore indicate fast changes in the recorded biosignals.

### Mean rise duration and STD rise duration

They compute vectors of the mean rise duration and the standard deviation over time.

### Rate

It calculates vectors of the heart, respiration and EDA rate over time.

#### d) The facial representation module

The facial animation module models the deformation of skin tissue according to a 3-layer model, consisting of skull, muscle and skin layers. Each layer consists of a number of nodes, which are connected with neighbouring nodes of the same layer and nodes in the layers above/below. Each node represents a mass and each link between nodes is modelled as a spring.

The module flow goes through several processing stages before producing the 3D reconstruction:

i) The features of the EMG signals, as extracted by the Feature Extraction Module, are used to estimate the contraction of the subject's monitored muscles. The outcome of this procedure is the quantification of muscle contraction for the sixteen muscles being monitored.

ii) The contraction level drives the muscle model, to calculate the new position of muscle-nodes. The muscle model is simulating linear and sphincter muscles, which are the kinds of muscles involved in AUBADE.

iii) Numerical methods, through the attachment of muscle nodes in the face's geometry, solve the mathematical model of the mass-spring network, given the new position of the muscle nodes.

iv) The displacement of each node of the skin mesh is then applied to the face's geometry, as calculated by the mathematical model in the previous step.

The generation of muscle force is computed by using integrated EMG as a measure of muscle activity, as follows:

The steady-state force M generated by muscle is

*M *= *k*_*f*_*SE*,     (3)

where *S *is the muscle cross-sectional area, *E *is the integrated EMG level normalised to a range between 0 (mean of baseline muscle activity) and 1 (maximum activity recorded, including a series of "maximal" facial gestures), and *k*_*f *_= 2500 dyne/cm2 is a scaling coefficient.

**Figure 3:**
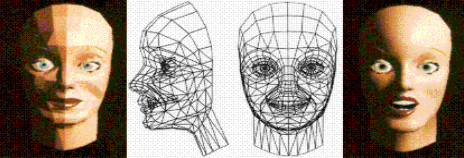
3-D generic model which is presented to the user.

The resulting 3D generic facial model is then presented on the user's screen as illustrated in Fig 3.

#### (e) The intelligent emotion recognition module

**Figure 4:**
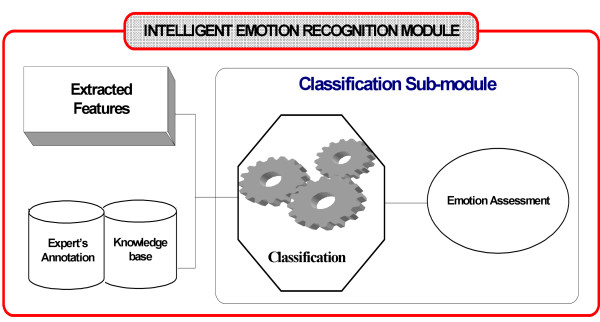
The Intelligent Emotion Recognition Module.

The Intelligent Emotion Recognition module is a decision support system that classifies the subject's basic emotions (into one of the pre-defined emotional classes) using the outcome of the feature extraction module. The module's schematic is presented in Fig. 4. The classification into predefined emotional classes was achieved using Support Vector Machines (SVM) [34,35]. Support Vector Machine is considered as a state-of-the-art classifier for both linear and non-linear classification. SVMs belong to the family of kernel based classifiers. SVMs implicitly map the data into the feature space where a hyperplane (decision boundary) separating the classes may exist. This implicit mapping is achieved with the use of kernels, which are functions that return the scalar product in the feature space by performing calculations in the data space.

Although initially developed for binary classification problems, SVMs can be adapted to deal with multi-class problems using the one-against-one method [36]. This method constructs *k*(*k*-1)/2 classifiers (where k is the number of classes) where each one is trained using data from two classes. Although other methods for multi-class SVMs exist, the above mentioned approach has been chosen due to the low training time required and its comparable performance [37].

#### f) AUBADE databases

The system's databases store the acquired raw signals which are ranked per user, per date, per event etc. They can be recalled any time from this database and can be analysed by specialists and researchers who are able to draw statistical and other information. The databases also store the medical history of the subjects as well as their facial animation videos.

## Results

After the extraction process where the abovementioned feature extraction algorithms are applied, vectors of the desired features are formed for each type of signal. A dataset is created containing the vector of extracted features along with the expert's annotation for every period of 10 s. This time window is a significant factor, for the output of the AUBADE intelligence module, since it determines how often it will provide updates about the emotional state of a user. The objective of a real time or near real time emotion classifier is to first recognize as correctly as possible the emotional state of the user (high classification rate), and second to recognize it as soon as possible (high sensitivity). The former suggests a large window size, to minimize variance in the features within a class. On the contrary, the latter suggests a small window size. The 10 second period window has been identified as the suitable compromise between these two arguments, based on the acknowledgment that there is a time delay between the instance that the subject experienced an emotion and the corresponding response changes in the selected biosignals [26].

The system has been validated using data obtained from four drivers in simulated race conditions. An experienced psychologist supervised the whole procedure and annotated each driver's emotional state every 10 s. The emotional classes identified were high stress, low stress, disappointment, euphoria and neutral face. The extracted vector of features along with the expert's annotation for every period of 10 s constituted the dataset for the classifier. The classification into predefined emotional classes was achieved using SVM with RBF kernel.

The feature extraction and the classification of the emotional state have been exhaustively tested and validated for driver #1. The methodology followed was: a whole race was used for the training of the classifier and a different race was used for testing it. The averaged results are presented in Table 2 in terms of Sensitivity and Positive Prediction Accuracy (PPA) which are defined as:

**Table 2:** Classification results

%	**High stress**	**Low stress**	**Euphoria**
Sensitivity	91.3	84.4	82.6
PPA	86.7	73.6	87.9
	**Disappointment**	**Neutral face**	**Classification Rate**
Sensitivity	79.3	92.4	**86.0**
PPA	90.9	93.3	

Sensitivityemotion_a=#oftemplatesclassifiedasemotion_aaccordingtoclassifiertotal# oftemplatesbelongingtoclassemotion_aaccordingtopsychologist×100%
 MathType@MTEF@5@5@+=feaafiart1ev1aaatCvAUfKttLearuWrP9MDH5MBPbIqV92AaeXatLxBI9gBaebbnrfifHhDYfgasaacH8akY=wiFfYdH8Gipec8Eeeu0xXdbba9frFj0=OqFfea0dXdd9vqai=hGuQ8kuc9pgc9s8qqaq=dirpe0xb9q8qiLsFr0=vr0=vr0dc8meaabaqaciaacaGaaeqabaqabeGadaaakeaaieaacqWFtbWucqWFLbqzcqWFUbGBcqWFZbWCcqWFPbqAcqWF0baDcqWFPbqAcqWF2bGDcqWFPbqAcqWF0baDcqWF5bqEdaWgaaWcbaGae8xzauMae8xBa0Mae83Ba8Mae8hDaqNae8xAaKMae83Ba8Mae8NBa4Mae83xa8Lae8xyaegabeaaiiaakiab+1da9maalaaabaGae83iamIae8hiaaIae83Ba8Mae8NzayMae8hiaaIae8hDaqNae8xzauMae8xBa0Mae8hCaaNae8hBaWMae8xyaeMae8hDaqNae8xzauMae83CamNae8hiaaIae83yamMae8hBaWMae8xyaeMae83CamNae83CamNae8xAaKMae8NzayMae8xAaKMae8xzauMae8hzaqMae8xyaeMae83CamNae8hiaaIae8xzauMae8xBa0Mae83Ba8Mae8hDaqNae8xAaKMae83Ba8Mae8NBa4Mae83xa8Lae8xyaeMae8hiaaIae8xyaeMae83yamMae83yamMae83Ba8Mae8NCaiNae8hzaqMae8xAaKMae8NBa4Mae83zaCMae8hiaaIae8hDaqNae83Ba8Mae8hiaaIae83yamMae8hBaWMae8xyaeMae83CamNae83CamNae8xAaKMae8NzayMae8xAaKMae8xzauMae8NCaihabaGae8hDaqNae83Ba8Mae8hDaqNae8xyaeMae8hBaWMaei4iamIaeeiiaaIae83Ba8Mae8NzayMae8hiaaIae8hDaqNae8xzauMae8xBa0Mae8hCaaNae8hBaWMae8xyaeMae8hDaqNae8xzauMae83CamNae8hiaaIae8NyaiMae8xzauMae8hBaWMae83Ba8Mae8NBa4Mae83zaCMae8xAaKMae8NBa4Mae83zaCMae8hiaaIae8hDaqNae83Ba8Mae8hiaaIae83yamMae8hBaWMae8xyaeMae83CamNae83CamNae8hiaaIae8xzauMae8xBa0Mae83Ba8Mae8hDaqNae8xAaKMae83Ba8Mae8NBa4Mae83xa8Lae8xyaeMae8hiaaIae8xyaeMae83yamMae83yamMae83Ba8Mae8NCaiNae8hzaqMae8xAaKMae8NBa4Mae83zaCMae8hiaaIae8hDaqNae83Ba8Mae8hiaaIae8hCaaNae83CamNae8xEaKNae83yamMae8hAaGMae83Ba8Mae8hBaWMae83Ba8Mae83zaCMae8xAaKMae83CamNae8hDaqhaaiabgEna0kab=fdaXiab=bdaWiab=bdaWiab=vcaLaaa@FB42@

PPA_emotion_a=#oftemplatescorrectlyclassifiedintoclassemotion_aaccordingtoclassifiersumoftemplatesclassifiedintoclassemotion_aaccordingtotheclassifier×100%
 MathType@MTEF@5@5@+=feaafiart1ev1aaatCvAUfKttLearuWrP9MDH5MBPbIqV92AaeXatLxBI9gBaebbnrfifHhDYfgasaacH8akY=wiFfYdH8Gipec8Eeeu0xXdbba9frFj0=OqFfea0dXdd9vqai=hGuQ8kuc9pgc9s8qqaq=dirpe0xb9q8qiLsFr0=vr0=vr0dc8meaabaqaciaacaGaaeqabaqabeGadaaakeaaieaacqWFqbaucqWFqbaucqWFbbqqcqWFFbWxcqWFLbqzcqWFTbqBcqWFVbWBcqWF0baDcqWFPbqAcqWFVbWBcqWFUbGBcqWFFbWxcqWFHbqyiiaacqGF9aqpdaWcaaqaaiab=ncaJiab=bcaGiab=9gaVjab=zgaMjab=bcaGiab=rha0jab=vgaLjab=1gaTjab=bhaWjab=XgaSjab=fgaHjab=rha0jab=vgaLjab=nhaZjab=bcaGiab=ngaJjab=9gaVjab=jhaYjab=jhaYjab=vgaLjab=ngaJjab=rha0jab=XgaSjab=Lha5jab=bcaGiab=ngaJjab=XgaSjab=fgaHjab=nhaZjab=nhaZjab=LgaPjab=zgaMjab=LgaPjab=vgaLjab=rgaKjab=bcaGiab=LgaPjab=5gaUjab=rha0jab=9gaVjab=bcaGiab=ngaJjab=XgaSjab=fgaHjab=nhaZjab=nhaZjab=bcaGiab=vgaLjab=1gaTjab=9gaVjab=rha0jab=LgaPjab=9gaVjab=5gaUjab=9faFjab=fgaHjab=bcaGiab=fgaHjab=ngaJjab=ngaJjab=9gaVjab=jhaYjab=rgaKjab=LgaPjab=5gaUjab=DgaNjab=bcaGiab=rha0jab=9gaVjab=bcaGiab=ngaJjab=XgaSjab=fgaHjab=nhaZjab=nhaZjab=LgaPjab=zgaMjab=LgaPjab=vgaLjab=jhaYbqaaiab=nhaZjab=vha1jab=1gaTjab=bcaGiab=9gaVjab=zgaMjab=bcaGiab=rha0jab=vgaLjab=1gaTjab=bhaWjab=XgaSjab=fgaHjab=rha0jab=vgaLjab=nhaZjab=bcaGiab=ngaJjab=XgaSjab=fgaHjab=nhaZjab=nhaZjab=LgaPjab=zgaMjab=LgaPjab=vgaLjab=rgaKjab=bcaGiab=LgaPjab=5gaUjab=rha0jab=9gaVjab=bcaGiab=ngaJjab=XgaSjab=fgaHjab=nhaZjab=nhaZjab=bcaGiab=vgaLjab=1gaTjab=9gaVjab=rha0jab=LgaPjab=9gaVjab=5gaUjab=9faFjab=fgaHjab=bcaGiab=fgaHjab=ngaJjab=ngaJjab=9gaVjab=jhaYjab=rgaKjab=LgaPjab=5gaUjab=DgaNjab=bcaGiab=rha0jab=9gaVjab=bcaGiab=rha0jab=HgaOjab=vgaLjab=bcaGiab=ngaJjab=XgaSjab=fgaHjab=nhaZjab=nhaZjab=LgaPjab=zgaMjab=LgaPjab=vgaLjab=jhaYbaacqGFxdaTcqWFXaqmcqWFWaamcqWFWaamcqWFLaqjaaa@0B1F@

## Discussion

The AUBADE system recognizes and estimates basic emotions in real-time, in the form of a "diagnosis". AUBADE is a multifunctional system that can be utilized in many different ways and in multiple application fields.

The system's clinical application is based on the ability of supporting clinical diagnosis related to all the pathologies according to which the patient's capability to feel and express emotions is limited or totally absent. In those cases, doctors need to know the physiological condition of their patients. This is achieved by recording the expressions of the patient's face. Thus, muscle spasms as well as skin conductivity measurements are of key importance. As far the medical domain, the system is applied in the following cases:

### Parkinson's disease

In general patients affected by Parkinson's disease lose their capability to express emotions and become inexpressive. AUBADE will be used on patients affected by Parkinson's disease at different stage of disease development (classified using Unified Parkinson's Disease Rating Scale), in order to assess the capability to express emotions.

### Stroke

Stroke deeply impacts emotional behaviour and Stroke survivors often show inappropriate emotions and extreme mood fluctuations. In particular, they may laugh when something isn't funny or cry for no apparent reason. AUBADE system will then be used to correlate emotions with the stage of disease.

### Huntington's disease (HD)

Patients with Huntington's disease show deficits in the recognition of anger and fear, and an especially severe problem with disgust, which was recognized only at chance level. Consequently, some neurologists are investigating if the same patients may be able of feeling and expressing disgust themselves.

### Cortical lesions

Cortical lesion influences expression; the monitoring of facial expression in patients under emotional solicitation can greatly assist in the diagnosis of:

▪ lesions of the supplementary motor area (medial part of the frontal lobe), which lead to contralateral facial paresis, with spontaneous emotional expression more affected than voluntary.

▪ lesions of the motor cortex (also with contralateral facial hemiparesis), which affect voluntary movements but leave intact spontaneous smiling.

▪ frontal lobe lesions, which lead to fewer spontaneous expressions of brow raising, smiling, lip tightening, tongue protrusion, etc. during neuropsychological testing of brain injured subjects.

The effects of biofeedback, used therapeutically for this condition could be tracked using AUBADE for facial expression analysis.

AUBADE will be used mainly to detect the absence of a specific emotion (in this case we are talking about a basic emotion according to FACS methodology) as well as the absence of every type of emotion (or the presence of a neutral face independently from the stimulus provided) as well as the presence of a "wrong" (not expected) emotion in response of a particular stimulus provided.

As far as the car racing domain, AUBADE will be a useful tool for the mechanics of car racings, because they will be able to monitor emotionally the users. Moreover the car's setting and development will not only be based in subjective questionnaires filled by the driver, but in driver's emotional state (fear, stress level), which straightly correlates with the car's performance. Finally, it may reduce accidents in car racings. Emotions and our psychological situation generally affect our behavior and reactions. Thus, if some emotion is detected that in some way may affect the behavior of the user, then the observer will be able to provide him with additional advices and guidance, preventing some reaction of the user that would be fateful.

AUBADE's classification accuracy into five predefined emotional classes is 86.0%. It must be noticed that the above results, although promising, are only indicative. The system will be extensively tested and evaluated on car racing drivers of Maserati, following all relevant Federal Insurance Administration (FIA) regulations and other European ethical directives in relation to privacy of personal data and secure transfer of medical information.

## Conclusion

A novel system that automatically monitors and classifies the psychological condition of human subjects from a set of emotions by applying pattern recognition techniques is presented. AUBADE estimates the emotional state of human subjects by classifying vectors of features extracted from: facial Electromyogram, Respiration, Electrodermal Activity and Electrocardiogram. It is designed to be applicable to persons operating under extreme stress conditions, such as car racing drivers. In the medical field, AUBADE may be effectively utilized for patients suffering from neurological and psychological disorders.

The usual way to assess human emotion is by employing advanced image-processing techniques in order to extract the facial characteristics. In our case, it is very difficult to apply image-processing techniques, since for safety reasons the users are wearing a mask and above it a casque. The proposed system realises an alternative method in order to record the facial expressions of the subject. Instead of using image-processing techniques, AUBADE utilizes the processing of surface EMG sensors, placed on the fireproof mask that the users are currently wearing.

A computational method for emotion recognition utilizing an SVM classifier is introduced. The method appears to have high performance both in terms of accuracy and computational efficiency. Due to the fact that emotions vary from person to person, the system must be trained using a variety of subjects and then tested for its performance.
